# Percutaneous versus thoracoscopic ablation of symptomatic paroxysmal atrial fibrillation: a randomised controlled trial - the FAST II study

**DOI:** 10.1186/s13019-018-0792-8

**Published:** 2018-10-03

**Authors:** Jesper Eske Sindby, Henrik Vadmann, Søren Lundbye-Christensen, Sam Riahi, Søren Hjortshøj, Lucas V A Boersma, Jan Jesper Andreasen

**Affiliations:** 1 Department of Cardiothoracic Surgery, Aalborg University Hospital, Hobrovej 18-22, 9000 Aalborg, Denmark; 2 Department of Cardiology, Aalborg University Hospital, Aalborg, Denmark; 3 Unit of Clinical Biostatistics, Aalborg University Hospital, Aalborg, Denmark; 4 Atrial Fibrillation Study Group, Aalborg University Hospital, Aalborg, Denmark; 5 Department of Cardiology, St. Antonius Hospital, Nieuwegein, The Netherlands; 6 AMC Amsterdam, University of Amsterdam, Amsterdam, The Netherlands

**Keywords:** Paroxysmal atrial fibrillation, Catheter ablation, Thoracoscopic, Randomised study

## Abstract

**Background:**

The most efficient first-time invasive treatment, for achieving sinus rhythm, in symptomatic paroxysmal atrial fibrillation has not been established. We aimed to compare percutaneous catheter and video-assisted thoracoscopic pulmonary vein radiofrequency ablation in patients referred for first-time invasive treatment due to symptomatic paroxysmal atrial fibrillation. The primary outcome of interest was the prevalence of atrial fibrillation with and without anti-arrhythmic drugs at 12 months.

**Methods:**

Ninety patients were planned to be randomised to either video-assisted thoracoscopic radiofrequency pulmonary vein ablation with concomitant left atrial appendage excision or percutaneous catheter pulmonary vein ablation. Episodes of atrial fibrillation were defined as more than 30 s of atrial fibrillation observed on Holter monitoring/telemetry or clinical episodes documented by ECG.

**Results:**

The study was terminated prematurely due to a lack of eligible patients. Only 21 patients were randomised and treated according to the study protocol. Thoracoscopic pulmonary vein ablation was performed in 10 patients, and 11 patients were treated with catheter ablation. The absence of atrial fibrillation without the use of anti-arrhythmic drugs throughout the follow-up was observed in 70% of patients following thoracoscopic pulmonary vein ablation and 18% after catheter ablation (*p* < 0.03).

**Conclusion:**

Thoracoscopic pulmonary vein ablation may be superior to catheter ablation for first-time invasive treatment of symptomatic paroxysmal atrial fibrillation with regard to obtaining sinus rhythm off anti-arrhythmic drugs 12 months postoperative.

**Trial registration:**

ClinicalTrials.gov Identifier: NCT01336075. Registered April 15th, 2011.

## Background

Atrial fibrillation (AF) is characterized by disorganized, rapid, and irregular contraction of the atria. Its effects on hemodynamic and thromboembolic events result in significant morbidity, mortality, impaired quality of life (QOL), hospitalizations, and health-cost. It is the most common sustained cardiac arrhythmia. By 2030, it is estimated that 14–17 millions Europeans will suffer from this arrhythmia, with 120,000 to 215,000 newly diagnosed patients per year [1].

Many patients are first diagnosed with AF when they are admitted to the hospital for AF related event (transient ischaemic attacks (TIA), stroke etc.). Other patients are increasingly affected by their symptoms (dyspnoea, palpitations etc.) with episodes increasing in severity and duration. AF is a progressive disease, where paroxysmal AF (PAF) can transform into persistent AF, long-standing persistent and permanent AF.

The current understanding of the pathophysiology of AF implies that ‘triggers’ or foci may be located in the pulmonary veins. Furthermore, electrical and structural changes of the atrium itself may serve as a ‘substrate’ that can perpetuate AF [1].

AF is treated medically with varying results and there is no definitive long-term curative treatment. The main goal aims at reducing symptoms and preventing disabling complications. Treatment normally includes antithrombotic, rhythm, and/or rate management. The decision regarding acute or long-term management depends on severity of the symptoms.

Non-pharmacological interventions have evolved over the last few decades to prevent and treat atrial fibrillation and/or to reduce symptoms. These interventions include catheter ablation (CA), video-assisted thoracoscopic (VATS) ablation and surgical Maze procedures. Surgical incisions or lines made by different energy sources, e.g., radio frequency or cryo-ablation, inhibit the progression of electrical impulses from spreading within the atrium [2].

The rationale for eliminating AF with ablation includes a potential improvement in quality of life [3], a decreased stroke risk [4] and a decreased heart failure risk and improved survival.

The long-term results of different treatments modalities are emerging. However, few randomised trials have been conducted to compare the surgical and CA modalities. The Atrial Fibrillation Catheter Ablation Versus Surgical Ablation Treatment (FAST) study randomized patients with previously failed CA to thoracoscopic pulmonary vein ablation (PVI) or repeat CA, which showed significantly greater efficacy of VATS PVI, but at the price of a significantly higher adverse event rate [5]. Other studies on surgical treatment have shown difference in efficacy depending on whether patients had paroxysmal, persistent, long-standing persistent or permanent AF and which lesion-set was made [6].

The most efficient first-time invasive treatment, for achieving sinus rhythm, in symptomatic paroxysmal atrial fibrillation has not been established. We aimed to compare the results of CA versus VATS pulmonary vein isolation (PVI) as a first invasive treatment in symptomatic paroxysmal AF patients. The primary outcome of interest was the prevalence of AF with and without anti-arrhythmic drugs (AAD) after 12 months.

## Methods

The study was designed as a dual-centre, prospective randomised study. Two centres were planned to include and allocate patients 1:1, i.e. the Departments of Cardiology and Cardiothoracic Surgery, Aalborg University Hospital, Denmark; and the Departments of Cardiology and Cardiothoracic surgery, St. Antonius Hospital, Nieuwegein, The Netherlands.

Enrolment began in April 2011 and was planned to end during the spring of 2013, or when the planned number of patients was enrolled. Due to administrative reasons and the relocation of surgical staff, no patients were included at St. Antonius Hospital.

Eligible patients referred to the Department of Cardiology, Aalborg University Hospital, Denmark, were screened, enrolled and randomised through an automatic digital call centre by cardiologists after oral and written informed consent from the patient was obtained.

The Danish Ethics Committee approved the study (VEK project ID: N20110009), and the trial was registered at ClinicalTrials.gov - Identifier: NCT01336075.

### Inclusion criteria

Eligible patients were patients with recurrent symptomatic paroxysmal AF. Inclusion criteria were previous failure with one or more AAD (treatment > 30 days) and/or cardioversion or any contraindications against treatment with AADs. ADDs were amiodarone, flecainid, propafenone, sotalol, beta-blockers and dronedarone.

Further inclusion criteria were willingness and ability to attend the scheduled follow-up visits, age between 18 and 75 years, and the provision of signed informed consent.

### Exclusion criteria

Exclusion criteria were persistent, long-standing persistent or permanent AF, a previous AF ablation procedure, AF secondary to electrolyte imbalance, thyroid disease, a reversible or non-cardiac cause, severe underlying heart disease (congenital heart disease, significant valvular disease, cardiomyopathy with a left ventricular ejection fraction (LVEF) < 35%, or angina pectoris/ischaemic heart disease), severe enlargement of the left atrium (> 45 mm), the presence of a pacemaker, failure to obtain informed consent, pregnancy or breastfeeding, an inability to undergo transesophegeal echocardiography (TEE), a documented left atrial thrombus, the presence of co-morbid conditions that, in the opinion of the investigator, constitute an increased risk for general anaesthesia or port access, e.g., pleural fibrosis, chronic obstructive pulmonary disease (Forced Expiratory Volume during 1 s. < 1.5 L/s), known internal carotid artery stenosis (> 80%), current enrolment in another clinical trial, life expectancy < 1 year, or previous transient ischaemic attacks (TIA)/stroke.

### Procedure

Both CA and VATS PVI are standard treatment options at Aalborg University Hospital. Approximately 200 CA procedures are performed annually by three cardiologists. One surgeon performed all VATS PVI procedures.

### VATS PVI

The procedure was performed under general anaesthesia with intravenous medication and the placement of a double-lumen endotracheal tube. TEE was performed in the operating room (OR) to verify the absence of a left atrial thrombus before the start of the operation. The procedure has been described in details by Wolf et al. [7] and Edgerton et al. [8].

Briefly, three ports were introduced on each side. The pericardium was incised from the superior vena cava to the inferior vena cava 2–3 cm anterior and parallel to the phrenic nerve. Blunt dissection around the pulmonary veins (PV) was facilitated by an articulated lighted dissector (Lumitip™ Dissector probe, AtriCure, Inc., Cincinnati, Ohio). Correct positioning of the ablation clamp (Isolator Synergy™ Clamp, AtriCure, Inc., Cincinnati, Ohio) on the atrium and not on the PVs was verified via direct inspection of the device after closing the jaws of the clamp. Bipolar RF energy was applied to electrically isolate the PVs; two to five overlapping lesions were created to ensure isolation. When the conductance of the tissue decreased to less than 0.0025 Siemens, an audible signal was automatically generated to indicate that the lesion was transmural. Stimulation with the Coolrail™ linear pen (AtriCure, Inc., Cincinnati, Ohio) on the PVs and atria confirmed conductance blockade of the area.

A chest tube was placed, the right lung was re-inflated, and the port sites were closed. The technique was repeated on the left side with the addition of division of the ligament of Marshall. The left atrial appendage (LAA) was then excised using a stapling device (EZ 45–60 stapler, Ethicon Endosurgery). LAA exclusion was verified on TEE. Pacing of the PVs during sinus rhythm was performed to ensure conductance blockade of the ablation-lines. No heparin was used during the procedure. If the patient was not in sinus rhythm by the end of the procedure, a synchronized direct-current shock was performed to establish sinus rhythm (SR). Extubation was performed in the OR.

### Percutaneous radiofrequency catheter ablation

Combined with computer tomography scan, a complete anatomical image of the left atrium was generated with the CARTO® mapping system (Biosense Webster Inc., Diamond Bar, CA, USA), allowing 3-D non-fluoroscopic navigation in the left atrium. The ablation procedure was performed as described extensively in the literature by Oral et al. [9] and Pappone et al. [10]. Briefly, access to the left atrium was achieved through a standard transseptal puncture using the Brockenbrough technique. Two 8F sheaths were advanced to the left atrium through two separate transseptal punctures. During the procedure, unfractionated heparin was administered to maintain an activated clotting time value > 300 s., measured every 30 min. Ablation was performed with a 4-mm irrigated tip catheter. Circumferential ablation lines were performed, encircling the left and right PVs in the left atrium, with a demonstration of electrical discontinuity between the PV and the atrium as an endpoint. Electrical discontinuity was demonstrated by means of a Lasso catheter placed in the PVs and/or by the mapping/ablation catheter.

### End points

The primary endpoint was freedom from AF/left atrial tachycardia without antiarrhythmic therapy at 6- and 12-month follow-ups as determined by 7 days of Holter monitoring, ECG, and patient interviews. AF recurrence documented by ECG, admission to the hospital due to AF beyond a blanking period from 3 to 12 months were also considered treatment failures in addition to re-intervention.

An episode of AF was defined as more than 30 s of AF observed on Holter monitoring/telemetry or clinical symptoms with ECG documentation of AF.

Secondary endpoints were symptom improvement by the European Heart Rate Association score (EHRA), the absence of AF/left atrial tachycardia with AADs, procedural complications (local haematomas/ecchymosis, thromboembolism, vasovagal reaction, haemothorax, pneumothorax, infection at the entry sites, endocarditis, pulmonary vein stenosis, cardiac perforation or tamponade, complete heart block, air embolism, arrhythmias, vascular damage or insufficiency, pericardial effusion, TIA, pericarditis, phrenic nerve damage, death, sternotomy, pain, pneumonia, or chest pain/discomfort), and reduction of the AF-burden.

Data were collected at follow-up visits at one, three, six and 12 months and from unplanned admissions to the hospital and outpatient clinic. Holter monitoring was performed for 7 days shortly before the follow-up visits at six and 12 months. Information on EHRA-score, medication, AF recurrence and complications were recorded.

### Statistical analysis

A power calculation was performed with a significance level of 5% and a power of 90% assuming success rates of 70% and 90% for percutaneous CA and VATS PVI, respectively. Therefore, the study required 79 patients in each arm. Assuming a dropout rate of 10%, the inclusion of 90 patients in each arm was planned.

All continuous variables were reported as means and standard deviations. Comparisons between groups were conducted using an unpaired t-test with a bootstrap calculation of standard error to address potential non-normality and variance inhomogeneity. Categorical variables were reported as numbers and percentages. Comparisons were conducted using Fisher’s exact test. EHRA scores over time were analysed using Wald test based on repeated measures model. Measures of associations with *p*-values (two-tailed) < 0.05 were considered statistically significant. STATA, version 13.1 (StataCorp, College Station, TX, US) was the statistical software used.

## Results

The study was terminated in January 2014 as only 21 patients had been enrolled at Aalborg University Hospital. No patients were included in the Dutch centre due to administrative reasons and due to relocation of the surgical staff. Termination of the study was due to a lack of progress in enrolment caused by a lack of patients who fulfilled the inclusion criteria, as well as administrative challenges. The first patient was screened in July 2011, and by November 2013, 209 patients had been screened for possible enrolment in the study.

**Fig. 1:**
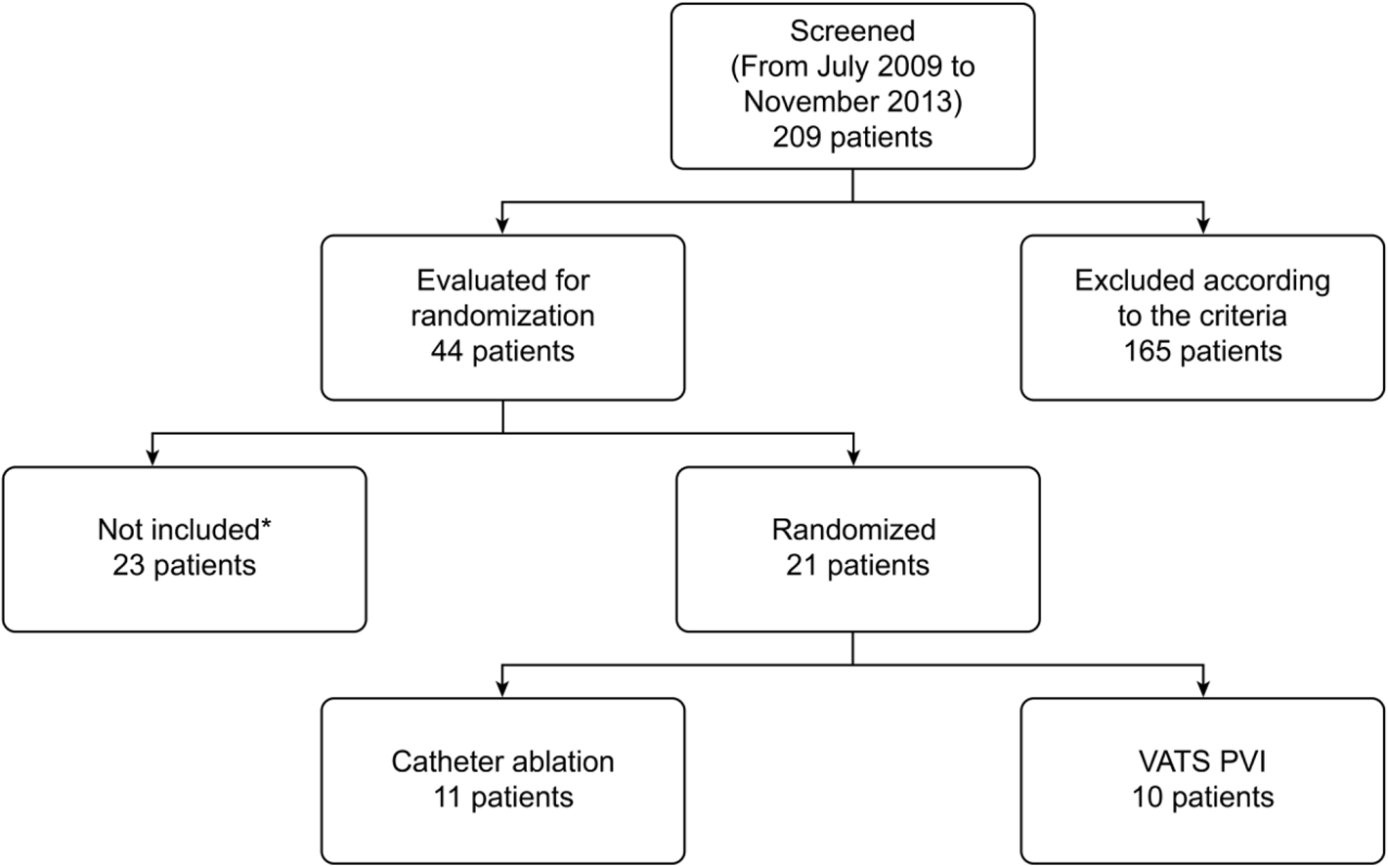
Flow chart of enrolment

Figure 1 shows the patient flow diagram.

Of the screened patients, 165 were excluded according to the criteria. Of the remaining 44 patients eligible for randomization, 22 patients declined participation after written and oral study information was provided. A total of 22 patients were randomized. One patient in the PVI group had a malignant looking nodulus on chest x-ray and crossed over to CA group after removal of a pT1aN0M0 adenocarcinoma from the lower right pulmonary lobe. One patient in CA group was excluded after the procedure as it turned out the patient had an underlying atrioventricular nodal re-entrant-tachycardia.

After 6 months of follow-up, one patient in the VATS PVI group declined further participation in the study.

Baseline patient characteristics are shown in Table 1.

**Table 1:** Patient characteristics

	CA	VATS PVI
Age at procedure – Years (Mean ± SD)	55.5 (8.1)	53.5 (6.7)
BMI (Mean ± SD)	27.8 (4.4)	28.1 (3.8)
LVEF % (Mean ± SD)	64.5 (4.7)	63.5 (7.1)
LA Diameter – mm (Mean ± SD)	42.2 (3.2)	42.3 (5.1)
DC-conversion pre proc. (Mean ± SD)	2.3 (3.0)	1.5 (3.2)
Sex (Male/Female)	8/3	9/1
Hypertension, n (%)	4 (36.4)	3 (30)
Symptoms - EHRA 2, n (%)	5 (45.5)	5 (50)
Symptoms - EHRA 3, n (%)	6 (54.5)	5 (50)
CHADS2 – score 0, n (%)	4 (36.4)	5 (50)
CHADS2 – score 1, n (%)	4 (36.4)	4 (40)
CHADS2 – score 2, n (%)	3 (27.2)	1 (10)
COPD, n (%)	0	0
Diabetes, n (%)	2 (18.2)	0 (0)
AAD - 0	0	1 (10)
AAD - 1	2 (18.2)	1 (10)
AAD - 2	6 (54.5)	3 (30)
AAD – 3	3 (27.3)	3 (30)
AAD - 4	0	0
AAD - 5	0	2 (20)

### Efficacy of CA and VATS PVI

**Fig. 2:**
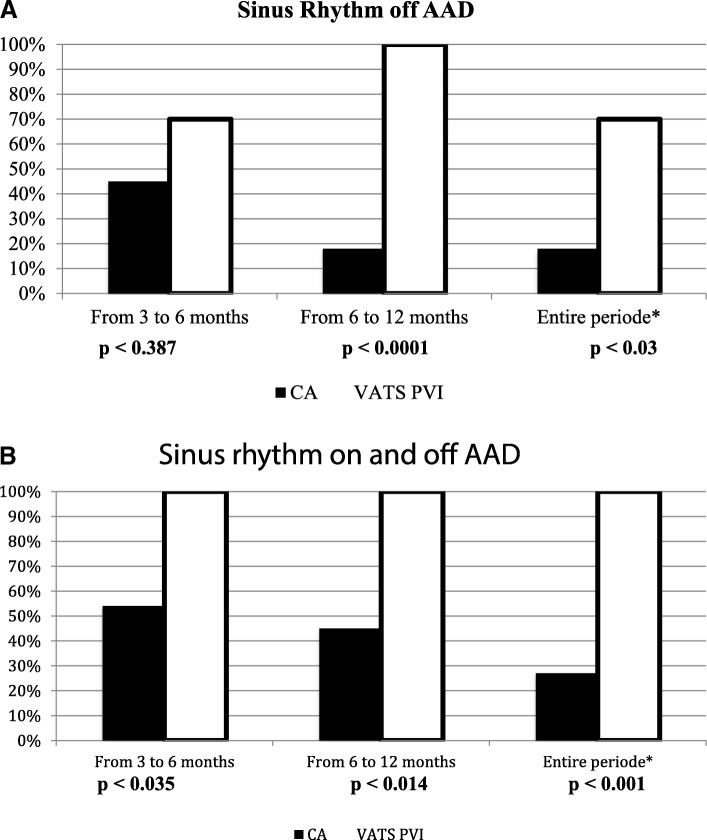
**a** Patients achieving SR off anti arrhythmic drugs. **b** Patients achieving SR on and off anti arrhythmic drugs

Figure 2a and b shows the success-rates with regard to being in SR at 3-to-6 month, 6-to-12 month and for the entire 3-to-12 month period after the procedure. Figure 2a shows patients off AAD. Figure 2b shows the combination of on and off AAD.

A total of 18% of the patients in the CA group and 70% in VATS PVI group (*p* < 0.03) achieved SR off AAD for the entire postoperative blanking period. For patients both on and off AAD the results were 27% versus 100%, respectively (*p* < 0.001).

The results from Holter monitoring after CA at six and 12 months showed 80% and 70% in SR +/− AADs, respectively. For VATS PVI, the results showed 100% in SR +/− AADs at 6 and 12 months.

At the 6-month follow-up, two patients lacked 7-day Holter monitoring data and two patients lacked Holter monitoring data at the 12-month follow-up. The Holter monitoring data at six and 12 months showed that two and three different patients respectively, all in the CA group, had documented AF.

No patients suffered a stroke, TIA or bleeding complications during the follow-up.

**Fig. 3:**
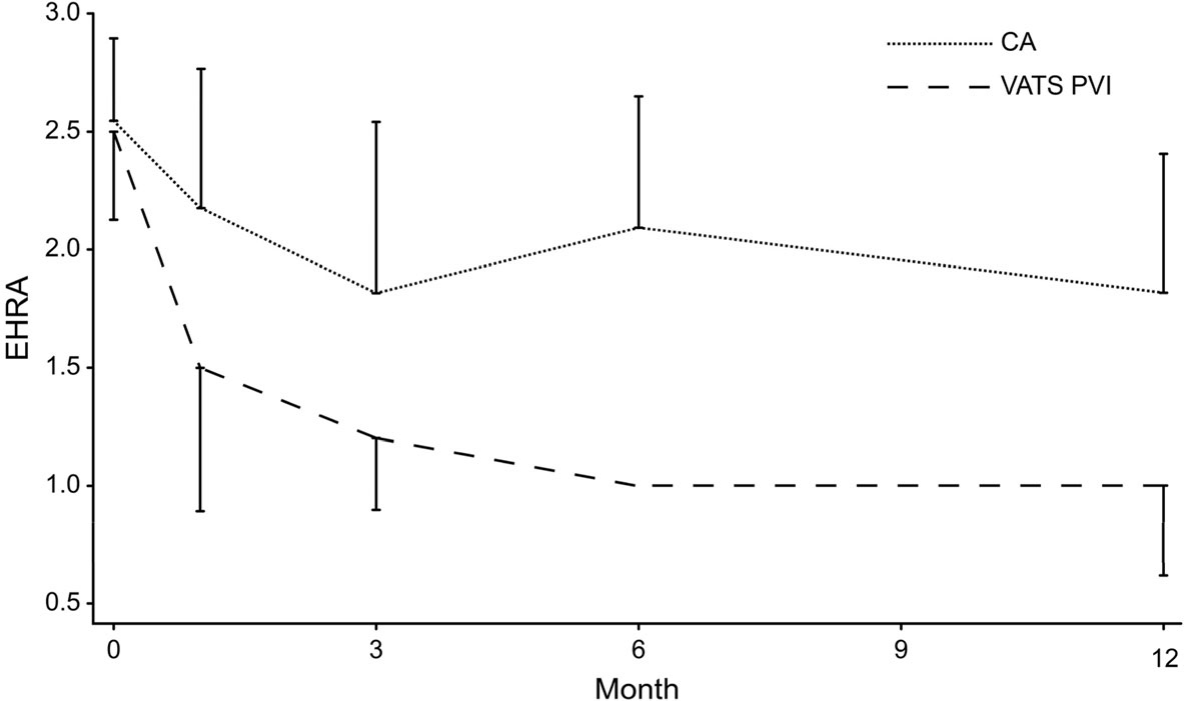
EHRA-score over time

Figure 3 depicts development of symptoms, classified in EHRA-score, from before the procedure to 12 month after.

### Safety of CA and VATS PVI (Tables 2 and 3)

**Table 2:** Procedure characteristics

	CA	VATS PVI
Procedure time	174 min	168.5 min
Grey/Radiation	28 Gy (Range 12–61)	0
Admitted – Days	1 (SD 0)	7.6 (Range 3–21)
Removal of LAA	0%	100%
Procedural endpoint achieved	100%	100%

*CA* Catheter Ablation, *VATS PVI* Video Assisted Thoracoscopic Pulmonary Vein Isolation, *Gy* Grey, *SD* Standard Deviation, *LAA* Left Atrial Appendage

**Table 3:** Complications

	CA	VATS PVI
Procedure complications	0	1^a^
Complications during hospitalization	0	2^b^
Complications 1 month	1^c^	0
Complications 3 month	0	0
Complications 6 month	1^d^	0
Complications 12 month	0	0

*CA* Catheter Ablation, *VATS PVI* Video Assisted Thoracoscopic Pulmonary Vein Isolation

^a^ The patient was converted to sternotomy due to bleeding. A full Maze IV was performed and the patient was discharged on post-operative day five without any further events; ^b^ One patient with heart block which resolved spontaneously and one patient with tamponade (relived with percutaneous drainage) and pneumonia; ^c^ Patient had discomfort in the chest and resolved within a few days without treatment; ^d^ Re-ablated and pacemaker insertion

## Discussion

In this randomised clinical trial, VATS PVI was compared with CA as a first-time invasive treatment for patients with paroxysmal AF. The results from this limited number of patients indicated that VATS PVI might be superior to CA for the first-time invasive treatment of paroxysmal AF relative to the primary endpoint of freedom from AF without AADs at 12 months. However, the VATS PVI procedure resulted in a higher rate of complications and an increased length of hospital stay. Due to the premature termination of the study, no solid conclusions can be drawn.

For the entire follow-up period, VATS PVI was significantly better at achieving SR without AADs.

The optimal follow-up would have been continuous monitoring of the heart rhythm for the entire period as suggested by The Society of Thoracic Surgeons [11]. We present the results from the different time periods along with the scheduled Holter monitoring at 6 and 12 month including the success-rate for the entire follow-up (3 to 12 month) as it somewhat resembles a continuous monitoring.

Our dataset is too small to assess reliable differences in success rates between the different time periods within the CA and VATS PVI groups. Most AF relapses occurred outside the scheduled Holter monitoring period. A 7-day Holter monitoring covers only a small portion of the follow-up period. Similar monitoring issues could be suspected when comparing results from the present study with other studies.

No pre-operative Holter monitoring was performed, so a comparison with 7-day Holter monitoring at six and 12 months could not be done. According to the Holter monitoring data, five patients showed various degrees of AF-burden (from 0.1 to 99.9%) in the CA group.

The Atrial Fibrillation Catheter Ablation Versus Surgical Ablation Treatment (FAST) study [5] randomized patients with previously failed CA to VATS pulmonary vein isolation (PVI) or repeat CA and showed significantly greater efficacy with VATS PVI, but at the cost of a higher adverse event rate. Their results showed absence of AF without AADs at 12 months at 36,5% for CA and 65,6% for VATS PVI. However, the patients in the FAST-study differed from the patients in the present study in that some had paroxysmal AF, persistent AF and/or an enlarged left atrium and previously failed CA. Patients with an enlarged atria and persistent AF especially, have lower success rates with PVI as the only treatment and could account for the failures in the FAST-study.

A recent review article on VATS PVI by Laar et al. [12] showed an efficacy rate of VATS PVI of 81% at 1 year (SR off AAD), which is similar to our results.

Different strategies for post-procedure monitoring have been applied in many studies and all have their strengths and weaknesses. Mostly, 1- to 7-day Holter monitoring has been used because it is relatively easy to apply and is patient-friendly. Unfortunately, it only covers a small fraction of the entire follow-up period. Pappone et al. [10] reported an average of 6 ± 4 AF episodes per month prior to treatment. A 24-h Holter monitoring would have an approximate 20% chance of detecting AF based on those numbers. Jaïs et al. [13] showed 4–30 episodes per month in which the average episode lasted 5.5 h (range 1–12 h). Therefore, Holter monitoring may have a very good positive predictive value but a low negative predictive value in this setting.

In a review article by Kis et al. on CA PVI treatment [14] showed a success rate of 78% (freedom from AF both on and off AADs) based on the majority of the included studies having 24-h Holter monitoring at different time intervals as a reflection of success. In our opinion, this would generally be insufficient to detect asymptomatic episodes of AF.

Because of different follow-up strategies, it is difficult to compare our CA results with others.

We suggest reporting data for the entire follow-up period and following patients for at least 1 year, preferably longer. Insertable Cardiac Monitor devices may be preferred in the future as they are becoming smaller and less expensive, and these devices can detect AF/Arrhythmia on a continuous basis for a longer period depending on the battery capacity [15]. However, there is risk of ‘noise’ and hence over-estimation of AF/arrhythmia.

Three out of five patients were reluctant to participate in the study, although the reason remained unspecified for the majority. Patients have different preferences and intentions when they come to the specialists; some choose for 100% efficacy, others choose for minimal impact and therefore they do not want to be randomized in a scientific study.

EHRA scores decreased over time in both groups, but improved more in the VATS PVI group than in the CA group, possibly due to the higher success rate of VATS PVI, even though CA has been shown to decrease the frequency and severity of symptoms [13].

The LARIAT-study showed a decrease in AF-burden after ligation of the LAA without any ablation [16]. This finding is of interest in relation to the present study as a major procedural difference between VATS PVI and CA was exclusion of the LAA. In a study by Di Biase et al. [17], it was shown that 27% of failed CA procedures occurred in patients with initiation/trigger points for AF in or around the LAA, which could partly explain the higher success rate with VATS PVI as the LAA is excluded. Romanov et al. [18] have found no difference in success rates when removing the LAA in patients with non-paroxysmal AF.

Ligation of the LAA as the only treatment [19] has shown a persistent decrease in blood pressure (BP) and a short-term reduction in sodium levels. Neuro-hormonal changes are still unclear, but indicate that AF could be much more than just a ‘mechanical’ problem of irregular heartbeats.

Concerning matters of LAA, one should notice that this study was not designed nor powered for a comparison of ablation lines but in order to compare two invasive treatment procedures.

Complications occurred more often in the surgical group as also shown by others authors [1, 5, 12]. The main reasons for prolonged hospital stays in the present study were pneumonia, temporary heart block and pain management. Complications in the VATS PVI group were dealt with during the hospital stay and none of the patients had problems after discharge. Laar et al. [12] showed a complication rate similar to CA after minor complications e.g. pneumothorax and pleural effusions were excluded from analysis.

Even though the objectives of the procedures are similar, the risk-profiles of CA and VATS PVI are different [1]. To optimize communication with patients, complications may be classified as life-threatening, severe, moderate, minor and unknown as outlined in published guidelines [1]. The risk-profile and long-term benefits of a procedure should be taken into consideration and conveyed to the patient when choosing a treatment strategy.

A major limitation of this study is the low number of patients included, and therefore the study was not powered for firm conclusions.

## Conclusion

VATS PVI may be superior to CA for the first-time invasive treatment of symptomatic paroxysmal AF in obtaining freedom from AF without AADs 12 months postoperative. However, a higher rate of complications and longer hospitalization is associated with VATS PVI. Although this was a randomised study, no solid conclusions can be drawn based on the results from this study due to the low number of patients included. A similar study should be carried out in centres with more patients.

## Abbreviations

AAD: Anti-arrhythmic drugs
AF: Atrial fibrillation
CA: Catheter ablation
EHRA: European Heart Rate Association score
LAA: Left Atrial Appendage
LVEF: Left ventricular ejection fraction
OR: Operating room
PV: Pulmonary veins
PVI: Pulmonary vein isolation
SR: Sinus rhythm
TEE: Transesophegeal echocardiography
TIA: Transient ischaemic attacks
VATS: Video-assisted thoracoscopic
